# Neutrophil extracellular traps regulating tumorimmunity in hepatocellular carcinoma

**DOI:** 10.3389/fimmu.2023.1253964

**Published:** 2023-12-18

**Authors:** Weixiong Zhu, Chuanlei Fan, Shi Dong, Xin Li, Haofei Chen, Wence Zhou

**Affiliations:** ^1^The Second Clinical Medical College, Lanzhou University, Lanzhou, China; ^2^Department of General Surgery, The Second Hospital of Lanzhou University, Lanzhou, China; ^3^The First Clinical Medical College, Lanzhou University, Lanzhou, China

**Keywords:** hepatocellular carcinoma (HCC), neutrophil extracellular trap (NET) formation, tumorigenesis and progression, liver transplantation, liver ischemia-reperfusion injury (liver I/R injury)

## Abstract

As a component of the innate immune system, there is emerging evidence to suggest that neutrophils may play a critical role in the initiation and progression of hepatocellular carcinoma (HCC). Neutrophil extracellular traps (NETs) are web-like chromatin structures that protrude from the membranes during neutrophil activation. Recent research has shown that NETs, which are at the forefront of the renewed interest in neutrophil studies, are increasingly intertwined with HCC. By exploring the mechanisms of NETs in HCC, we aim to improve our understanding of the role of NETs and gain deeper insights into neutrophil biology. Therefore, this article provides a summary of key findings and discusses the emerging field of NETs in HCC.

## 1 Introduction

Primary liver cancer is the third leading cause of cancer-related death worldwide (1). Hepatocellular carcinoma (HCC), the most common form of primary liver cancer, often develops in the setting of chronic liver disease and cirrhosis (2, 3). The progression of HCC involves the evasion of immune surveillance and the establishment of an immunosuppressive tumor microenvironment (TME), thereby inhibiting cytotoxic immune cells (4). HCC is characterized by a high infiltration of leukocytes from multiple immune cell lineages, which negatively affects effector lymphocyte activity and correlates with poor outcomes (5–7). Therefore, the current focus in HCC research lies not only on lymphocytes but also on exploring the interactions of multiple immune cells (4).

Neutrophils, one of the phagocytes, as the first line of defense against pathogen invasion by employing their potent antimicrobial arsenal and also contribute to the activation of adaptive immunity (8, 9). In addition, neutrophils play a critical role in tumor progression and tumorigenesis through the release of various mediators. Tumor-related inflammation, in contrast to conventional inflammation, promotes tumor initiation and growth by facilitating the evasion of immune surveillance (10). Neutrophils, as representatives of conventional inflammation, show distinct effects on the malignant phenotype of tumors, as supported by numerous lines of evidence (4, 11, 12).

Previous studies have focused primarily on the role of neutrophils as pathogen scavengers during conventional inflammation (13). However, it is now recognized that neutrophils have multiple and diverse functions (13). In humans, neutrophils represent 50-70% of all circulating leukocytes, whereas in mice they make up 10-25% (14, 15). Notably, neutrophils play a crucial role in chronic inflammation, specifically in chronic liver disease and malignancies, facilitating immune infiltration (16, 17). The challenge at hand is that tumor immunotherapy has shown limited efficacy in many patients with HCC, which represents a significant obstacle in the field (18–21). For certain subgroups of liver cancer patients, a combination of targeted therapy and immunotherapy targeting infiltrating immune cells (except for T lymphocytes), may prove more effective than immune checkpoint inhibitors such as atezolizumab plus bevacizumab. By modulating the functional characteristics of neutrophils, it may be possible to enhance the sensitivity of HCC patients to systemic therapy by altering the immune microenvironment (4).

In addition to producing oxidants, proteins, and granular enzymes, neutrophils also have the ability to generate NETs (9, 22, 23). NETs consist of aggregated DNA that serves as a backbone that interacts with various molecules either positively or negatively (24). By forming NETs, activated neutrophils can ensnare a wide range of microorganisms, including bacteria (24, 25), fungi (26, 27), and viruses (28–31), enabling an effective response and subsequent clearance through the action of effector molecules (23). Numerous studies have provided evidence for the involvement of NETs in tumor initiation, progression, and angiogenesis (32–34). Interestingly, NETs also play a similar role in HCC (35, 36).

In this article, we review evidence for the presence of NETs in HCC which may support the hypothesis that NETs play a pivotal role in the pathogenesis of liver cancer. We discuss the proposed mechanisms by which NETs may drive tumor progression. Furthermore, considering that research on NETs in HCC is still at an early stage, we examine studies conducted on NETs in other types of cancers and discuss emerging areas of interest.

## 2 NETs discovery, origin and diversity

### 2.1 The historical and current existence of NET

NET formation, the process responsible for the formation of NETs to eliminate invaders through the release of granular proteins and chromatin, can be classified into two subtypes: lytic and vital (24, 37). Lytic NET formation, also known as suicidal NET formation, involves a slow, active cell death that occurs over several hours, distinguishing it from other forms of cell death such as necrosis or apoptosis (37). In addition to suicidal NET formation, another rapid process known as “vital NET formation”, which rapidly expels DNA from the nucleus or mitochondria of living cells within a few minutes while maintaining cell functionality and viability.

**Figure 1:**
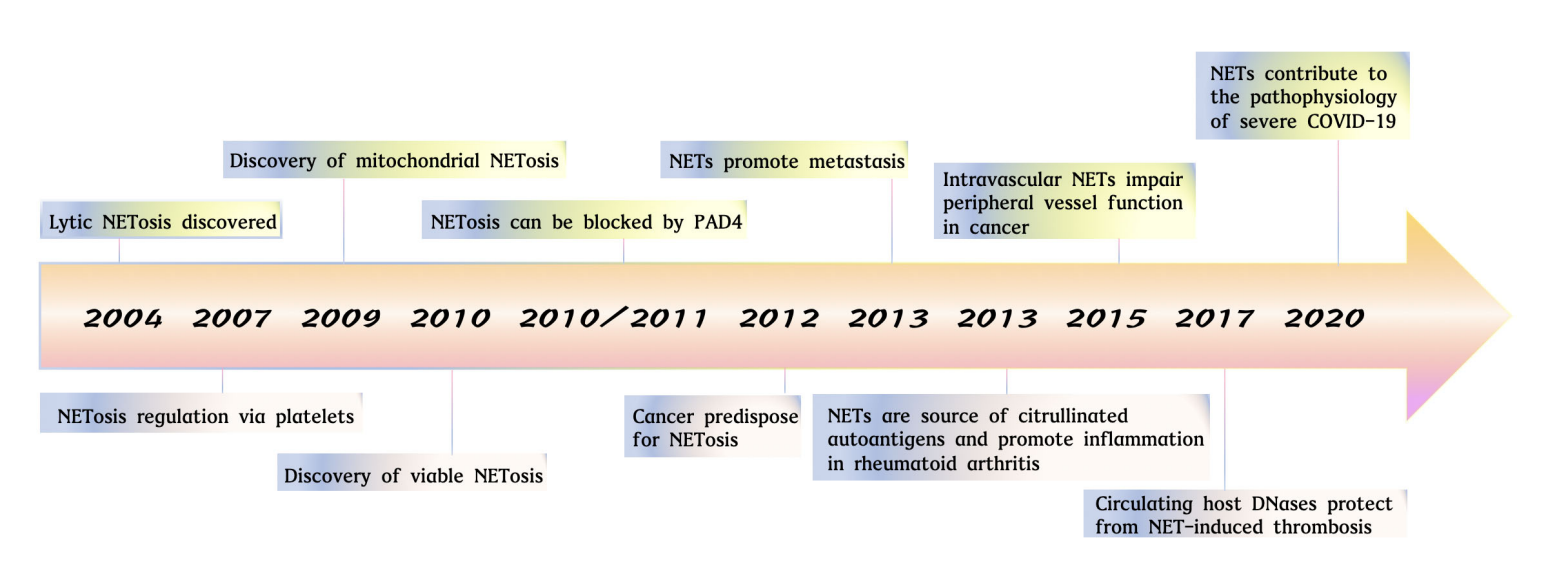
The studies of neutrophil extracellular traps from 2004 to 2021. Key observations in studies on NET formation are highlighted. Lytic NETosis discovered (24). NETosis regulation via platelets (42). Discovery of mitochondrial NETosis (23). Discovery pf viable NETosis (43). NETosis can be blocked by PAD4 (44, 45). Cancer predispose for NETosis (46). NETs promote metastasis (47). NETs are source of citrullinated autoantigens and promote inflammation in rheumatoid arthritis (48). Intravascular NETs impair peripheral vessel function in cancer (49). Circulating host DNases protect from NET-induced thrombosis (50). NETs contribute to the pathophysiology of severe COVID-19 (31).

Phorbol 12-myristate 13-acetate (PMA), a classic nonphysiological stimulus, induces a distinct form of neutrophil death that is fundamentally different from apoptosis and necrosis (38). PMA promotes cell death by increasing chromatin loosening and incorporating the nuclear envelope into organelles, a process that occurs within a few hours (39, 40). This process involves four key steps: (1) enhanced plasma membrane permeability, (2) accelerated disintegration of the nuclear envelope and cytoskeleton, (3) assembly of antimicrobial proteins onto chromatin scaffolds, and (4) decondensation of chromatin (41). *In vitro* experiments using isolated neutrophils treated with PMA have provided valuable mechanistic insights into lytic NET formation (**Figure 1**), demonstrating that neutrophil activation is accompanied by the assembly and activation of a multicomponent nicotinamide adenine dinucleotide phosphate (NADPH) oxidase and ROS (51–53). ROS can be generated by both NADPH oxidase and mitochondrial metabolism (53). In addition, G protein-coupled receptors (GPCRs) (54), CXC chemokine receptors (CXCRs) (55), toll-like receptors (TLRs) (56, 57), and cytokine receptors contribute to this process. Hydrogen peroxide (H_2_O_2_) is a robust oxidant generated by NADPH oxidase during the reduction of molecular oxygen through electron transfer, and it plays a crucial role in both NET and ROS generation (41). The correlation between neutrophil metabolism and ROS generation has been highlighted in mechanistic studies (58, 59). In NADPH oxidase-deficient neutrophils, PMA compensates for the lack of NADPH oxidase and, in combination with protein kinase C (PKC), triggers calcium release and subsequent activation of the Raf-MEK-ERK pathway (60)and ROS-dependent p38MAPK (61). However, in mice, NET formation may not be associated with ROS generated by NADPH oxidase (62). In addition to the classical pathway requiring ROS, two recent papers reported a novel pathway of NETosis mediated by a pore-forming protein, gasdermin D (GSDMD), that is independent of ROS (63, 64). Actin cytoskeletal dynamics plays a critical role in neutrophil activation, and during the first half hour after stimulation, limiting actin polymerization reduces NET formation (65).

Peptidyl-arginine deiminase 4 (PAD4), an intracellular calcium-dependent enzyme, activated by intracellular calcium levels. It converts arginine to citrulline. Upon translocation to the nucleus, PAD4 deaminates histones H2A, H3, and H4, resulting in decreased chromatin compactness (66, 67). Notably, mouse model studies indicate that NET formation explicitly relies on PAD4 and neutrophil elastase (NE) (62). However, recent research suggests that PAD4 is mainly required for NET formation in response to ionomycin and immune complexes, whereas it is dispensable for NET formation induced by cholesterol crystals, fungi, or PMA in human neutrophils (44, 68, 69). Inhibition of PAD2, another member of the PAD family, can reduce NET formation and inflammatory cytokine production (45). NE, which is released from intracellular granules into the cytoplasm during NET formation, may then degrade linker histones to promote chromatin decondensation. Interestingly, NE and myeloperoxidase (MPO), released from azurophilic granules and translocated to the nucleus, cooperate in facilitating chromatin decondensation independently of their enzymatic activities (70, 71).

In 2009, pivotal research identified a distinct form of NET formation known as vital NET formation, which is distinct from lytic NET formation. It is induced by granulocyte-macrophage colony-stimulating factor (GM-CSF), followed by stimulation with lipopolysaccharide (LPS) or C5a, and can occur either a ROS-dependent or independent manner (23, 72, 73). The entire process of vital NET formation proceeds by a unique and remarkably rapid mechanism, that takes only 5-60 minutes and being independent of oxidants (72). Notably, the existence of lytic NET formation upon LPS treatment suggests that different modes of NET formation under different circumstances synergistically contribute to immune defense (74). Importantly, there is heterogeneity in the propensity of neutrophils to undergo NET formation, with studies reporting a range of 10% to 60% of cells capable of NET formation at a given moment (37, 70, 75, 76).

At particular times in their lifespan, a subset of neutrophils expresses Olfactomedin 4 (OLFM4) and CD177, two molecular markers associated with NET biology, regardless of their maturation or activation stage (77, 78). Approximately 25% of circulating neutrophils in healthy individuals express OLFM4 [73]. This expression of OLFM4 in a subset of neutrophils is conserved in mice, and it is secreted and colocalized with NETs (79, 80). Clinical studies suggest that high numbers of OLFM4^+^ neutrophils in patients with septic shock are associated with risk of organ failure and death (81). This is consistent with the findings that OLFM4^-^ neutrophils, in contrast to their OLFM4^+^ counterparts, provide protection against sepsis-induced mortality in OLFM4^-/-^ mice following LPS stimulation (79). These studies suggest that OLFM4 may reflect NET-induced toxicity rather than serve as a marker of NET formation. Another neutrophil protein associated with NET formation is CD177, which is expressed by approximately half of the neutrophils in peripheral blood. Research has shown that CD177^-^ neutrophils do not respond to LPS stimulation via NET formation (82, 83). Therefore, the regulation of OLFM4 and CD177 in NET formation may be a promising avenue for therapeutic intervention in NET-associated diseases, whether inflammatory or non-inflammatory.

Recently, a significant discovery had been found in the process of NETosis (84). They found that NETosis is triggered by mitogens and results in the upregulation of cell cycle markers, such as the phosphorylation of retinoblastoma proteins and laminin, disruption of the nuclear membrane, and replication of centrosomes. Further investigations revealed that CDK6, and possibly CDK4, are necessary for the signaling of NETosis. Interestingly, inhibition of CDK4/6 did not affect ROS production, leading to the hypothesis that CDK might function downstream of ROS activation. On the other hand, the translocation of NE to the nucleus relies on the activity of CDK4/6. However, the connection between CDK activity and ROS activation remains unclear, particularly since CDK6 also translocates to the nucleus during NETosis (84). CDK activation during NETosis occurs downstream of MAPK, although further experiments are required to confirm this. Future studies should focus on identifying the substrates of CDK4/6 involved in the NETosis pathway, as well as the mechanisms enabling neutrophils to respond to CDK4/6 activation, ultimately resulting in cell death instead of cell proliferation. Notably, investigating the role of CDKs in neutrophils using transfection or gene editing is challenging due to the short lifespan of neutrophils in culture (1-2 days). However, Amulic et al. ingeniously synthesized a peptide that mimics the inhibitory structural domain of p21 associated with CDKs (85). This peptide was coupled with a cell-penetrating sequence, providing an innovative approach for researchers facing difficulties in studying neutrophils due to their short half-life.

### 2.2 NETs content

**Figure 2:**
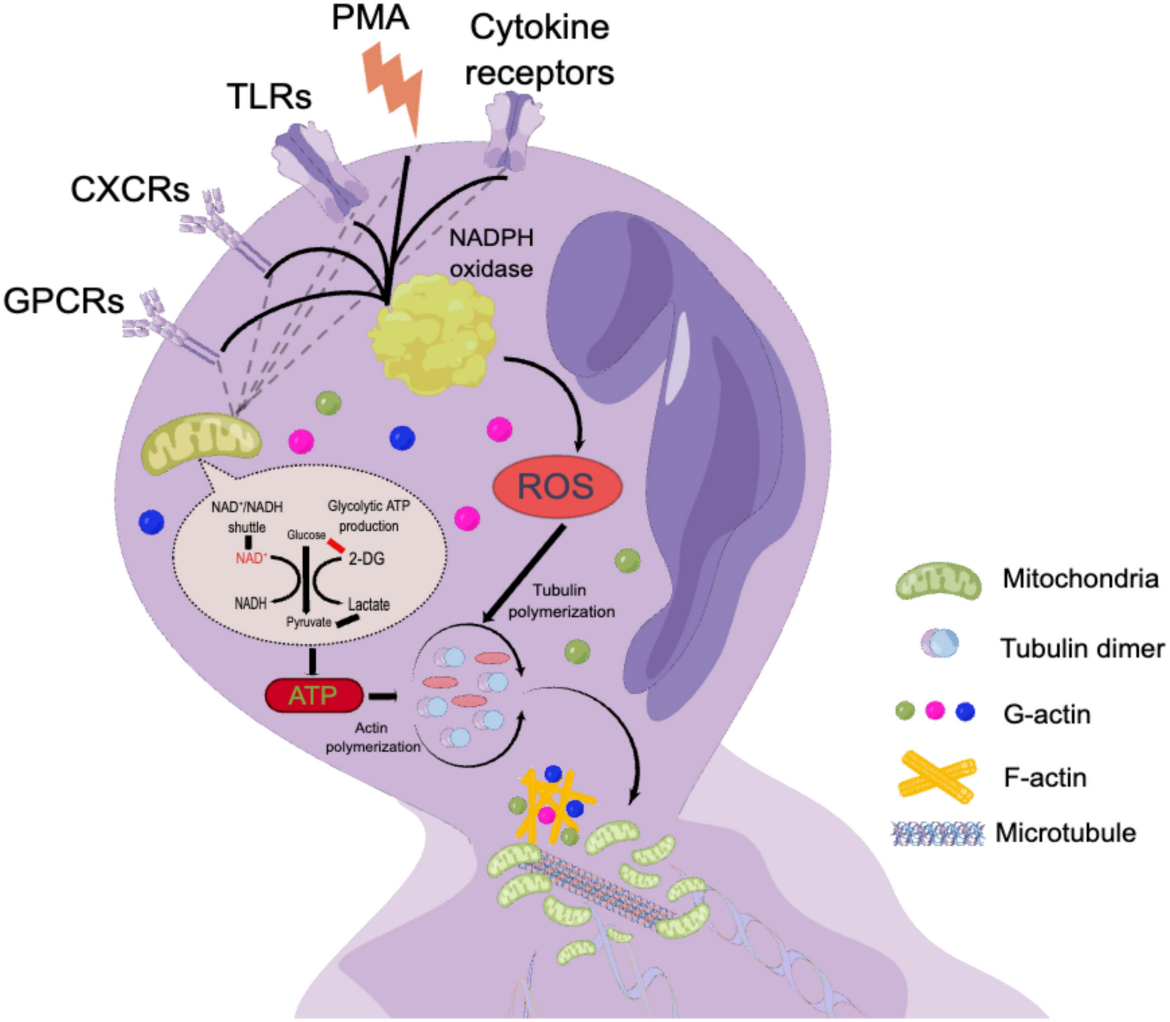
The mechanism of lytic NET formation. By processing isolated neutrophils with PMA in neutrophil, which in turn lead to the production of multicomponent NADPH oxidase and ROS (52–54). ROS can be produced by either NADPH oxidase or mitochondrial metabolism (54). GPCRs (55)、CXCRs (56)、TLRs (57) and cytokine receptors were also involved in this process.

The structure of NETs consists primarily of loosely packed chromatin composed of DNA and RNA, together with proteins composed predominantly of positively charged histones (86). The positive charge of these proteins facilitates the adsorption of small negatively charged foreign particles, while histones and nucleic acids themselves have bactericidal effects (87). In addition to histones, NET-associated proteins include cytoplasmic, granule, and cytosolic proteins, as well as neutrophil-derived metabolic enzymes (70, 88). Previous studies have shown that NETs not only contain their own effector molecules but also trap and release cytokines from other cells (89). Due to the diverse environmental conditions, both the structure and proteome of NETs exhibit considerable variation (52, 86). However, the core NET proteome, primarily derived from neutrophils, consists of histones, NE, proteinase 3, MPO, α-defensins, and cathepsin G (90, 91). Therefore, it is essential to identify disease-specific NETs (**Figure 2**).

### 2.3 Neutrophil and NETs

**Figure 3:**
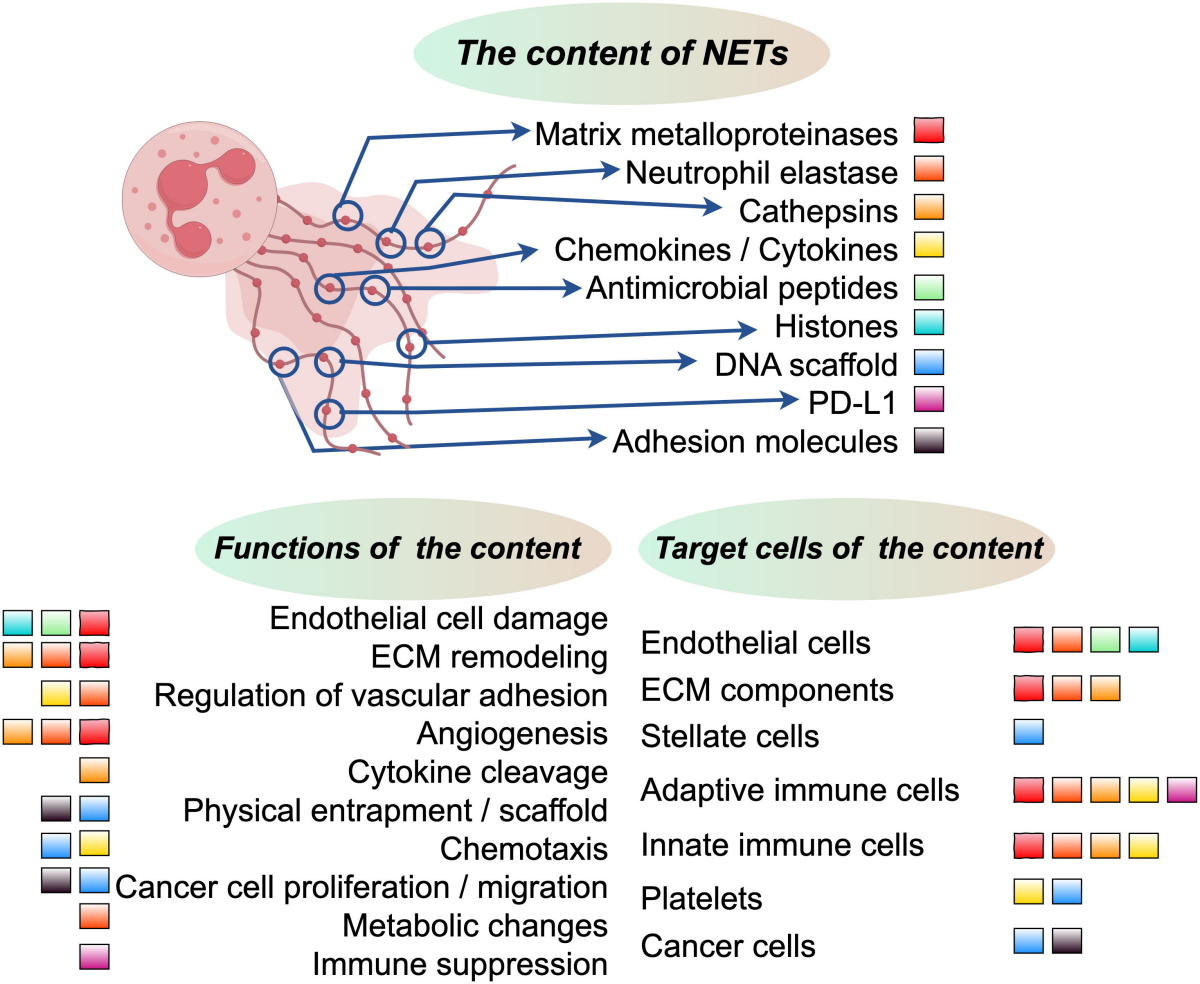
The upper section in the middle illustrates the core constituents of NETs, with each component represented by colored squares. The lower left section delineates the functional allocation of these core NET components, while the lower right section elucidates the distribution of cells or components that are targeted by these pivotal NET components.

A growing body of research suggests that not all neutrophils possess the ability to secrete NETs, which have been observed in diverse tissues, organs, and species. It is becoming increasingly clear that the molecular properties of NETs secreted by neutrophils varies across different stages of neutrophil maturation and among different neutrophil subtypes. Traditionally, neutrophils have been viewed as a homogeneous population of cells, but recent studies have revealed their plasticity, allowing them to adapt their phenotype to the environment and fulfill different functional requirements (11, 13). This adaptability is exemplified by their differential capacity to release NETs. The significant involvement of NETs in disease pathogenesis is underscored by a considerable increase in research efforts over the past decade (**Figure 3**).

Recent studies have shown that Only about 30% of mice neutrophils produce NETs whereas up to 60% of human neutrophils can produce NETs (70, 75). These observations suggest that not all types of neutrophils exhibit the same ability to release NETs under identical stimulation, though the underlying reasons for these differences remain unclear. Previous research has indicated that the increased capacity for NET formation corresponds to the transition from the immature stage to the mature stage in bone marrow-derived neutrophils of mice (92). However, circulating neutrophils, while still capable of releasing NETs, exhibit a reduced capacity for NET formation during their immature stage (93). The variation in the ability of neutrophils to generate NETs at different stages of disease in different tissue environments remains unclear and requires further investigation. It remains to be determined whether there are changes in the components of NETs under these circumstances. Investigation of such changes may provide insight into the initiation of NETs in different diseases and the subsequent progression of these diseases.

NET formation is subject to circadian regulation that is influenced by the contents of neutrophil, including proteolytic enzymes, antimicrobial peptides, and adhesion molecules (94). Intriguingly, degranulation has been observed under both acute activation and steady-state conditions. In support of this notion, CXCR2-mediated autocrine signals can induce degranulation, with mouse neutrophils exhibiting reduced content of primary granules during daylight hours after being mobilized into the circulation at night (55). Temporal degranulation which is tightly regulated by the circadian machinery, suggests a significant temporal dependence of NET formation, specifically within the 24-hour cycle. This finding is particularly relevant as recent studies have demonstrated remarkable discrepancies in NET formation *in vivo* for neutrophils isolated at different times, observed in both mice and humans, and replicated in cases of acute lung injury or liver ischemia-reperfusion injury (53). Disturbed circadian patterns have been linked to several diseases, including tumors (95–97). However, the mechanisms underlying the association between NETs and circadian pattern-related diseases are not yet fully understood, and further research is required in this area. It is also unclear whether NETs can serve as a clinically reliable marker to predict the impact of circadian pattern disruption on the development of specific diseases.

Recent studies have highlighted the critical role of the microbiome in regulating neutrophil aging (98). In inflammatory conditions, an overactive subset of aged neutrophils significantly enhances NET formation (98). Aging neutrophils, despite limited evidence for circadian regulation, exhibit a bias toward activation and NET formation in the context of stress-induced inflammation and vascular occlusion in mouse models of sickle cell disease (98–100). The quantity of NETs associated with Haemophilus sp. is elevated in the sputum of patients with chronic obstructive pulmonary disease (101). Accordingly, elimination of gastrointestinal bacteria in mice reduces NET formation; nevertheless, this strategy may have side effects (98). It is important to note that the absence of gut microbiota is correlated with increased NET formation in mouse models of acute mesenteric ischemia-reperfusion injury (102). Based on these observations, it can be concluded that the composition of the microbiome may have different effects on NET formation. Segmented filamentous bacteria promote NET formation in neutrophils, whereas in human neutrophils, Lactobacillus rhamnosus strain GG, a member of the probiotic system, inhibits NET formation by attenuating ROS production *in vitro* (98, 103). Interestingly, researchers have discovered that microorganism-derived metabolites can influence NET production in multiple and potentially deleterious ways. The regulation of NET production may depend on diurnal variations in the location, function of the microbiome, and the control of intestinal permeability (104).

The regulation of NET formation at different age and maturation stages is an important aspect to consider. A recent study has demonstrated that neutrophils egress from the bone marrow follows a circadian rhythm, with peak levels occurring in the morning (100). This observation implies that particular subsets of neutrophils, rather than only neutrophils of a certain age, are able to produce NETs. In addition, circadian fluctuations drive increased neutrophil output from the peripheral blood into tissues at night through the regulation of chemokine CXCL2 expression by the circadian clock gene Bmal1 and subsequent CXCR2-dependent signaling (100). Interestingly, NET formation in neutrophils also follows circadian rhythms, with recently released neutrophils being more susceptible to these rhythms. The Bmal1-CXCL2-CXCR2 axis plays a crucial role in NET formation, and any disruption to its components will impede this process (55). Another study found age-related variability in the ability of neutrophils to generate NETs, which may explain the increased susceptibility of the elderly to infection and age-related inflammation (105). This diminished capacity for NET formation was observed not only in mice (average age of 18 months) but also in humans (average age of ~70 years) (106, 107).

These factors may be attributed, among others, to the age-related increase in neutrophils within the bone marrow and the degranulation of circulating neutrophils. These processes have been linked to a decline in the capacity for NET formation (55). Aside from age, the distribution of neutrophils can also influence NET formation and exhibit significant associations with various diseases. In conditions such as sterile neutrophilia and septicemia, intravascular NETs can form clots in the circulation, obstructing blood vessels and causing organ damage in the absence of host DNases (50, 108). Similarly, the significant susceptibility to thrombosis and inflammatory injury in the liver and lungs may be attributed to the intensive ability of relatively immature neutrophils, which have not been completely cleared from the circulation, to produce NETs (49, 55, 109). In the context of persistent inflammation, neutrophil stimulation at various locations may lead to excessive NET formation, as observed in the lungs of SARS-Cov-2 patients (43), autoimmune diseases (52, 110), and mouse models of atherosclerosis (111, 112).

## 3 NETs in hepatocellular carcinoma

**Figure 4:**
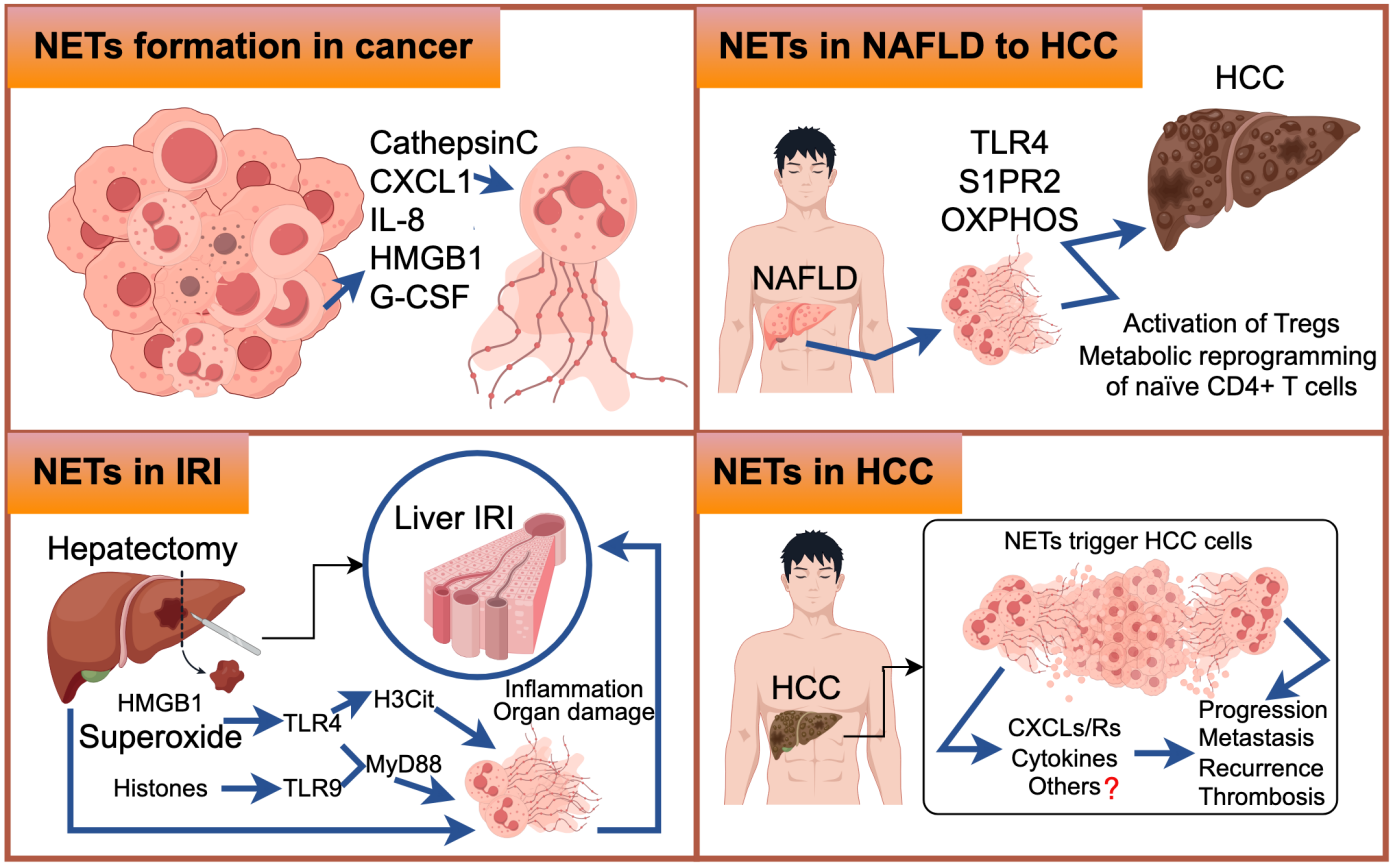
The upper-left figure illustrates selected molecular mechanisms underlying the formation of Neutrophil Extracellular Traps (NETs) in cancer. Similarly, the upper-right figure elucidates the molecular pathways through which NETs are generated during the transition from Non-Alcoholic Fatty Liver Disease (NAFLD) to Hepatocellular Carcinoma (HCC). In the lower-left figure, we delineate the molecular events that lead to NET formation in the context of Ischemia-Reperfusion Injury (IRI). The lower-right figure comprehensively outlines the intricate molecular processes associated with NET formation in HCC, with question marks indicating areas necessitating further exploration.

HCC typically begins insidiously and progresses rapidly. Unfortunately, by the time it is diagnosed, the majority of cases have already progressed to the intermediate and late stages, which are often associated with a grim prognosis. It has been observed that an elevated neutrophil-lymphocyte ratio (NLR) and a high infiltration of tumor-associated neutrophils are associated with worse outcomes in HCC (5, 7, 113). Nevertheless, the precise mechanisms through which neutrophils, particularly NETs, contribute significantly to the progression of HCC have yet to be thoroughly investigated. Apart from their well-known functions in innate immunity, NETs also play a crucial role in various stages of tumor initiation and progression. They promote tumor angiogenesis and growth, facilitate tumor expansion, and provide a protective shield for tumor cells against antitumor immunity (76, 114, 115). Notably, high expression of NETs has been observed in both the blood serum and tissue specimens of HCC patients, as well as in individuals with liver diseases such as nonalcoholic steatohepatitis and liver cirrhosis. This suggests a potential involvement of NETs in the development and progression of HCC (35, 46). Nevertheless, the specific molecular mechanism of NETs in HCC have yet to be fully elucidated. This review aims to summarize and provide further insights into these functions (**Figure 4**).

### 3.1 NETs in HCC initiation and growth

**Figure 5:**
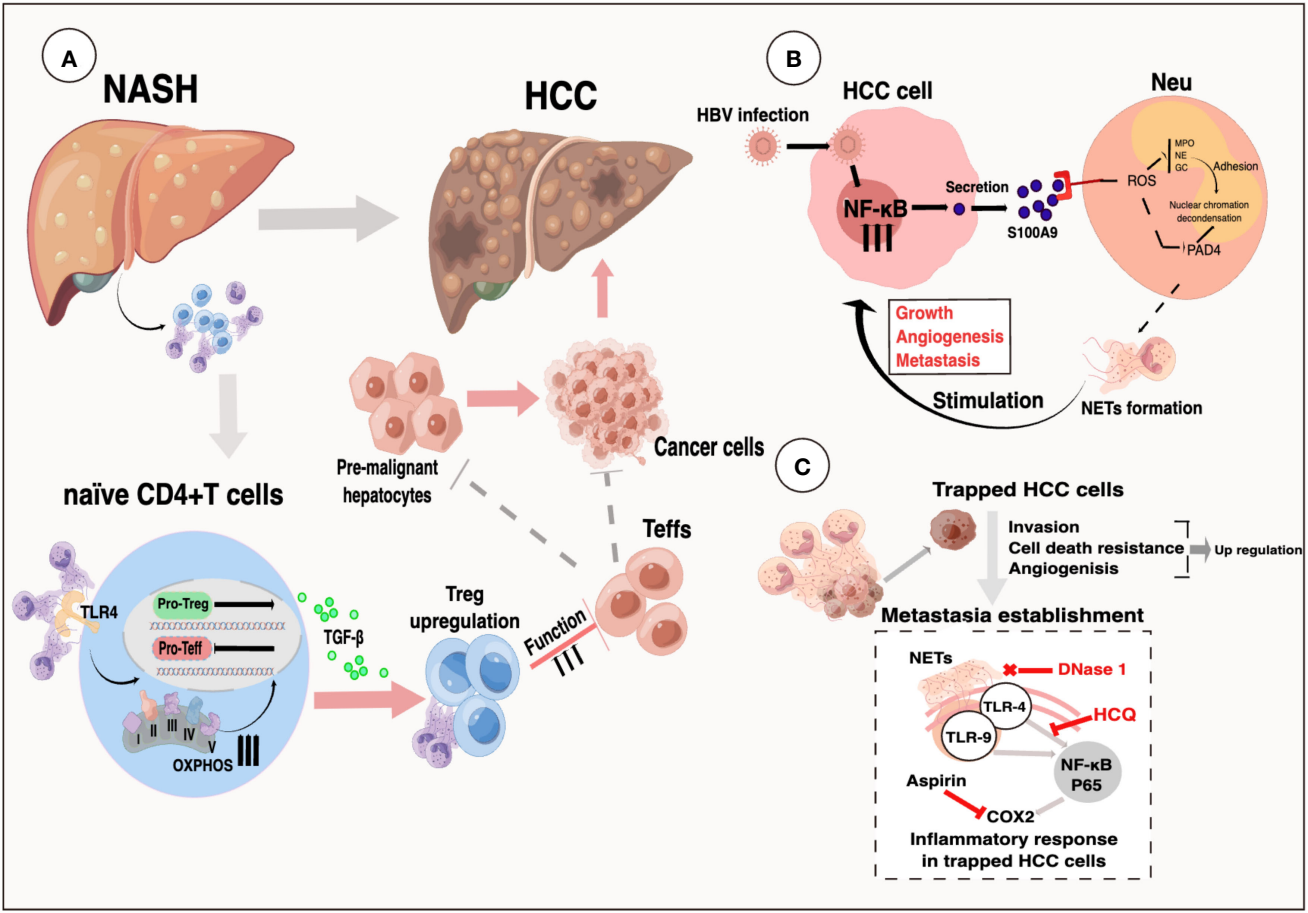
**(A)** Selectively increased intrahepatic Tregs can promote an immunosuppressive environment in NASH livers. NETs link innate and adaptive immunity by promoting Treg differentiation via metabolic reprogramming of naïve CD4^+^ T-cells (119). **(B)** Activation of RAGE/TLR4‐ROS signaling by HBV‐induced S100A9 resulted in abundant NETs formation, which subsequently facilitated the growth and metastasis of HCC cells (116). **(C)** System illustration of NETs to trap and fuel HCC metastasis potential through triggering an inflammatory response through TLR4/9-COX2 signaling (35).

Non-alcoholic fatty liver disease (NAFLD) encompasses a range of chronic hepatic disorders characterized by the accumulation of fat in hepatocytes, known as steatosis. It is commonly associated with obesity, hyperlipidemia, and insulin resistance. Notably, more than 10% of NAFLD patients progress to nonalcoholic steatohepatitis (NASH) (116), which represents the most significant risk factor for HCC development (117). In the livers of STAM mice (induced by neonatal streptozotocin and a high-fat diet), early infiltration of neutrophils and the formation of NETs were observed, followed by the production of inflammatory cytokines, the influx of monocyte-derived macrophages, and the progression of HCC (36). NETs formation, triggered by S1P receptor 2 signaling, accelerates inflammation and maintains disease progression in the early stages of NASH (118), as well as playing a crucial role in the transition from NASH to HCC and HCC metastases in later stages (36). It is worth noting that while the accumulation of fat in liver cells is independent of NET formation, NETs have an initiating role in the pathogenesis of fully developed NASH by modulating the inflammatory environment and promoting the activation of novel monocyte-derived macrophages (36). Treatment with DNase therapy or the use of PAD4^-/-^ mice did not affect the progression of hepatic steatosis but altered the subsequent hepatic inflammation pattern, ultimately resulting in reduced tumor growth (36). Inhibiting NETs *in vivo*, either through PAD4-deficient mice or treatment with DNase I, leads to reduced activity of regulatory T cells (Tregs) in NASH, and depletion of Tregs significantly inhibits the initiation and progression of HCC (119). RNA sequencing data revealed that NETs have an impact on gene expression profiles of naïve CD4^+^ T lymphocytes, particularly genes involved in oxidative phosphorylation in mitochondria (119). Enhancing mitochondrial respiration can promote the differentiation of regulatory T cells through the facilitation of NET formation (119) (**Figure 5A**). Future research should focus on investigating the specific components of NETs’ structure that interact with TLR4 on CD4^+^ T cells, as well as elucidating the properties of downstream signaling pathways. Moreover, further *in vivo* studies are necessary to understand the effect of NETs on Treg suppressive function. Targeting the interaction between neutrophils and Tregs or inhibiting Treg activity may enhance cancer immunosurveillance and prevent HCC formation. In HBV-infected HCC patients, NETs have been found to promote HCC proliferation both *in vitro* and *in vivo*, primarily through NETs-mediated cell entrapment, EMT-associated cellular migration, and MMPs-induced degradation of the extracellular matrix (ECM) (46).

The presence of NETs in the inflammatory microenvironment associated with liver cancer promotes tumor growth (120). *In vitro* experiments neutrophils from HCC patients showed a higher tendency to release NETs (35). A study revealed that plasma NET formation levels are increased in patients with liver cirrhosis and/or HCC, and these levels correlate with the severity of hepatic dysfunction (121). Elevated levels of MPO-DNA (NET serum markers) were associated with increased mortality after hepatectomy in HCC patients (122). There is growing evidence, albeit preliminary, suggesting that NET formation plays a crucial role in HCC development and proliferation. However, further research is needed to investigate the specific mechanisms of NET formation in HCC recurrence after surgical intervention, portal vein tumor thrombus, and systemic therapeutic resistance.

Liver transplantation offers benefits to patients with partial liver cancer. However, the occurrence of liver ischemia-reperfusion injury (IRI) is strongly associated with procedural failure in liver transplantation (123). Inflammation plays a significant role in the pathogenesis of liver IRI, with neutrophils being a key component of hepatic injury after reperfusion. Excessive activation and recruitment of neutrophils in the reperfusion tissue contribute to the development of ischemia-reperfusion injury. Neutrophils perform various functions throughout this intricate process, including activation, transport through the vasculature, and migration (124–126). A growing body of research has indicated that NETs could contribute to liver IRI. Released from damaged liver cells, HMGB1 proteins are associated with tissue damage and can trigger NET formation through the activation of TLR4 and TLR9 (127). In a study, extracellular superoxide treatment induced the release of NET DNA, citrullination of histone H3 (H3Cit) (128), and elevated levels of MPO-DNA complexes in neutrophils in a TLR-4 dependent manner. Pretreatment with allopurinol and N-acetylcysteine attenuated the formation of NETs and liver injury following ischemic injury in mice (56). Both neutrophils and NETs negatively correlate with the expression of histidine-rich glycoprotein (HRG) in mouse models of liver IRI. Overexpression of HRG in mice suppressed neutrophil infiltration and NET formation in the liver, thus reversing hepatic IRI (129). Another study demonstrated that hydroxychloroquine inhibits NET formation and alleviates liver IRI in mouse models of liver ischemia-reperfusion (48). Acrolein triggers neutrophil chemotaxis and exacerbates NET release both *in vitro* and *in vivo*. Inhibition of acrolein-induced NET release was observed *in vitro*, while suppression of inflammatory cytokine expression, P38MAPK-ERK activation, and apoptotic signals in the liver of mice subjected to IR resulted in a slower recovery rate of the liver after surgery (130). TMP, a compound derived from the plant Ligusticum wallichii Franchat, inhibited NET formation in rats after hepatic transplantation, improved hepatic function, and alleviated liver IRI (131). These studies have shown that antioxidant treatment can reduce NET formation and protect against hepatic IRI. However, when considering the therapeutic efficacy of inhibiting NETs, it is important to take into account the underlying preoperative disease and potential complications in immunocompromised individuals following transplantation.

### 3.2 NETs in hepatocellular carcinoma on the metastatic cascade

The previous study discovered that NETs sequester tumor cells in circulation and promote metastasis (132). Recently, researchers revealed that patients with liver cancer, particularly those with metastatic hepatocellular carcinoma, exhibit enhanced NET formation in neutrophils (133). NETs ensnare HCC cells, inducing resistance to cell death and enhancing invasiveness, thereby triggering metastatic potential. This process is mediated by the internalization of NETs into trapped HCC cells and activation of the TLR4/9-COX2 signaling pathway. In mouse models, a combination therapy involving DNase 1-mediated degradation of NETs and anti-inflammatory drugs such as aspirin/hydroxychloroquine effectively mitigates liver cancer metastasis (35). In HBV-infected HCC patients, the promotion of HCC metastasis by NETs was demonstrated both *in vitro* and *in vivo*, primarily through NETs-mediated cell entrapment, cell migration associated with epithelial-mesenchymal transition (EMT), and the degradation of ECM induced by MMP (46). Moreover, the induction of S100A9 by HBV accelerates the generation of NETs through the activation of TLR4 and the receptor for advanced glycation end products (RAGE), along with the signaling cascade involving ROS. Furthermore, the formation of NETs in the circulation was significantly associated with TNM stage and metastasis in HBV-induced liver cancer, and the presence of NETs was shown to have predictive value for extrahepatic metastasis (46) (**Figure 5B**). Deficient DNASE1L3 (an extracellular DNase) facilitates HCC invasion through NET formation by activating GMP-AMP synthase in circulation and the non-canonical pathway of NF-kappa B in diabetic HCC (47). Elevated levels of NET release have been observed in patients diagnosed with HCC, particularly those with portal vein tumor thrombosis (PVTT) (134). IL-8 produced by liver cancer cells triggers the formation of NETs via an NADPH oxidase-dependent pathway, and NET-associated cathepsin G (cG) facilitates hepatocellular carcinoma metastasis both *in vitro* and *in vivo* (134). The metabolic shift of tumor-associated neutrophils towards glycolysis and the pentose phosphate pathway, induced by tumors, promotes the formation of NETs in a ROS-dependent manner. Subsequently, NETs trigger tumor cell migration and suppress tight junction molecules on adjacent endothelial cells, thereby promoting tumor intravasation and metastasis (133). Activated neutrophils play a crucial role in the generation of ROS through their oxidative bursts (135). CXCL1-triggered hepatic impairment was found to be dependent on p47 phox, which mediates oxidative burst and is a constituent of the NADPH oxidase 2 complex (136, 137). In mice fed a high-fat diet, the absence of PAD4 or NE genes failed to abolish the capacity of elevated CXCL1 levels to induce NASH. This suggests that NETs or NE are not indispensable for the onset of CXCL1-induced NASH (138).Neutrophils in patients with liver cancer exhibit elevated levels of mitochondrial ROS (mitoROS) and generate NETs that are enriched with oxidation products of mitochondrial DNA (mtDNA) in a manner dependent on mitoROS (139). NET formation and oxidized mtDNA have clinical relevance. When bound to NET-associated proteins, oxidized mtDNA exhibits a heightened ability to elicit inflammatory mediators that promote metastasis in HepG2 cells (139). Targeting oxidized mtDNA with metformin can reduce the inflammatory state that facilitates metastasis, thereby undermining the metastatic capacity of hepatocellular carcinoma (139). Furthermore, high levels of NET infiltration have also been observed in patients with colon and breast cancers who have developed hepatic metastasis. Additionally, the level of NETs in serum can serve as a predictive marker for the incidence of liver metastases in early-stage breast cancer patients (17).

Previous research has revealed that soluble factors derived from tumors, such as hyaluronan fragments, metabolites, and cytokines, can have significant effects on the regulation of phenotypes and functions in tumor-infiltrating neutrophils. However, it is still uncertain which of these components, or if any other factors in the model, could be related to triggering the metastatic switch and leading to the release of NETs. In addition, DNase I has commonly been employed to eliminate NETs. However, a lingering question remains regarding whether DNase I eliminates the entire molecular structure of NETs or selectively targets specific components within the molecular structure. This uncertainty raises concerns about its potential impact on the specific mechanisms underlying NETs in HCC. Therefore, further investigations are warranted to address these inquiries thoroughly.

### 3.3 The role of NETS on hepatocellular cancer recurrence

Metastasis is the primary cause of carcinoma mortality, and neutrophils play a critical role in this process (140). Several studies have shown that NETs capture circulating tumor cells and release proteases, leading to tumor proliferation and metastasis (62, 141, 142). The capture of NETs has been associated with an increased incidence of microscopic liver metastases within 48 hours and a higher burden of gross metastatic disease at 2 weeks after tumor cell inoculation (132). One study suggests that elevated NET formation is linked to shorter recurrence-free survival and overall survival, making it a potential pre-surgery biomarker in serum for identifying patients at higher risk of recurrence (143). Another study demonstrated that HCC harbors NETs characterized by the enrichment of oxidized mitochondrial DNA, which promotes inflammation and metastasis (139). Enhanced circulating NET markers are also associated with an increased risk of recurrence in patients with advanced colorectal cancer and liver metastasis who have undergone major hepatic resection (34). Further study has found that NET formation may play a pivotal role in impeding emboli during metastasis, protecting them from NK cell attacks while traversing the bloodstream and infiltrating affected organs (144). Subsequently, another study combined this with engineering techniques: the application of an injectable hemostatic gel adhesive containing a neutralizer and NETs lyase has been shown to enhance adoptive natural killer cell immunotherapy and prevent liver cancer recurrence after resection (145). Additionally, NETs may act as a protective barrier, shielding cancer cells from the cytotoxicity of CD8^+^ T cells and NK cells. However, NETs are not the sole factor contributing to the impediment of antitumor immunity mediated by T cells and NK cells (144). The aforementioned studies indicate a positive correlation between elevated levels of NETs and an increased likelihood of HCC recurrence. However, the precise role of NETs in the specific mechanisms of HCC recurrence remains unclear. Additionally, it is yet to be determined whether the influence of NETs on HCC recurrence is influenced by circadian regulation, patient age, preoperative neoadjuvant therapy, postoperative chemotherapy, or other interactions that may impact HCC recurrence differently. Therefore, further investigations are needed to explore in-depth the specific mechanisms underlying the involvement of NETs in HCC recurrence.

### 3.4 The role of NETs in hepatocellular carcinoma on thrombosis

NETs are widely recognized for their crucial role in the pathogenesis of thrombosis and coagulation disorders, particularly in cancer (115, 146). Heparins, anticoagulant polyanions released by mast cells at pathogen entry sites, modify fibrin structure and stabilize fibrin clots through size-dependent modulation and kringle-dependent inhibition of plasmin-mediated fibrinolysis. All polyanions mechanically fortify the clots; however, smaller P45, P100, and low molecular weight heparin (LMWH) decrease the pliability of fibrin, while larger UFH and P700 increase the maximum tolerable deformation of blood clots (147). Administration of LMWHs inhibits the activation-induced autophagy of neutrophils and NET formation. Research has shown that LMWHs profoundly alter the capacity of neutrophils to produce NETs and mobilize primary granule contents in response to non-related inflammatory stimuli, such as HMGB1, PMA, and IL-8 in humans (148). Additionally, research has found that heparin and polyphosphate variants, procoagulant polyanions released by platelets and microorganisms, modulate the susceptibility of fibrin-histone clots to lysis and their mechanical stability. The size and charge of these variants play a crucial role in this process (42). Lu et al. developed a micellar nanoparticle loaded with doxorubicin, LMWH, and astaxanthin (LMWH-AST/DOX, LA/DOX NP) to prevent the occurrence of these loops and inhibit hepatic metastasis by inhibiting NET formation. These nanoparticles exhibited dual effects by not only inhibiting metastasis to the lungs but also mitigating the inflamed and immunosuppressed microenvironment within tumors (149).

Research has demonstrated that the interplay between neutrophils and platelets is especially significant in driving the formation of NETs and maintaining the process of diffuse coagulation (150–152). While this concept is substantiated in the context of sepsis, it necessitates further exploration in the context of HCC. This is especially pertinent for patients with HCC combined with portal vein thrombosis, as well as for those experiencing postoperative complications related to disseminated intravascular coagulation (DIC) in the context of HCC. Moreover, neutrophils primarily induce the formation of NETs through serine proteases, thereby activating both extrinsic and intrinsic coagulation pathways (109). A study investigating hepatic surgery observed that NETs and platelet-rich microthrombi contribute to microvascular alterations in injured organs. However, inhibiting NETs formation using DNase reduced immunothrombosis and organ damage (153). Another study demonstrated that the combined action of tissue plasminogen activator (tPA) and DNase I effectively triggers thrombolysis (154). Administering DNase I to rats with thrombosis proved successful in preventing myocardial infarction, recurrent cerebrovascular accidents, and deep vein thrombosis (155). Nevertheless, further research is needed to determine whether the degradation of NETs by DNase I could increase the risk of thrombosis and inflammation.

### 3.5 Approaches to targeting NETs in hepatocellular carcinoma

NETs have emerged as key contributors to the pathogenesis of various diseases, prompting extensive research into potential therapeutic targets. The presence of NETs has been linked to cancer patient survival (156, 157). However, it is crucial to carefully assess the risks associated with targeting NET formation. While NETs offer protection against severe infectious diseases, inhibiting NETs could potentially increase susceptibility to infections (44, 158). For instance, mice lacking the PAD4 gene (-/-) exhibited heightened vulnerability to bacterial diseases due to impaired NET formation, in contrast to their PAD4(+/+) counterparts (44). Conversely, a separate study found that PAD4 deficiency had no discernible impact on polymicrobial sepsis with concurrent bacteremia and the mitigation of endotoxemic shock (159). Notably, tumor cells, shielded from cytotoxicity by NET formation, play a pivotal role in the successful metastasis of cancer in mice. Moreover, the combination of PAD4 inhibitors and immune checkpoint inhibitors has demonstrated immunotherapeutic synergy by inhibiting the process of NET formation (144). Therefore, the risks associated with targeting NETs as therapeutic interventions may vary depending on the presence of specific disorders and the immunological state of the organism. Furthermore, the degradation of NET formation presents another significant hazard, as it leads to the release of DNA and histones derived from NETs, which have the potential to trigger inflammation. Currently, therapies aimed at modulating NET formation can be categorized into two distinct groups: inhibition of NET formation and destabilization/degradation of existing NETs.

Extensive research has already been conducted on the inhibition of NET formation. Inhibiting the expression of PAD4 can prevent NET formation (160). Another study demonstrated that suppressing the enzymatic function of PAD4 disrupts NET formation in both mice and humans, although it does not seem to affect bacteremia in the context of polymicrobial sepsis (161). Recent investigations have identified BMS-P5, GSK199, and GSK484 as inhibitors of NET development, capable of suppressing associated diseases in both *in vivo* and *in vitro* settings (161, 162). NE inhibitors, which hold great promise as therapeutic targets, play a pivotal role in suppressing NET formation (132). For instance, sivelestat, an NE inhibitor, was found to suppress NET formation in mice (163). Additionally, antibodies have also been recognized for their ability to impede NET formation. One such antibody, tACPA, restrains the development of NETs in neutrophil-mediated inflammatory conditions (164). However, the approach of targeting pre-existing NETs in HCC has not been thoroughly explored. NETs play a crucial role in the pathogenesis of HCC, and focusing on inhibitors or modulators of NETs in HCC may introduce a novel therapeutic approach for managing this malignancy.

An alternative approach to target NETs involves inducing their degradation. DNase I has been shown to possess the ability to partially lyse NETs (154). The suppression of NET production by DNase I effectively eliminates the promotion of HCC growth and metastasis induced by NET formation (46). In a murine model, the direct destruction of NET formation by DNase I, along with the administration of agents possessing anti-inflammatory properties such as hydroxychloroquine or aspirin, effectively attenuates liver cancer metastasis (35). Compelling evidence demonstrates that neutrophil-derived NET formation impedes the cytolytic activity of both cytotoxic T lymphocytes and NK cells during their interactions with cancer cells (144). Moreover, the presence of NETs and an acidic tumor microenvironment significantly counteract the potential benefits of cancer therapy involving NK cell infusion.

In experimental model, we consistently observed a concurrent occurrence of autophagy and the formation of NETs. The use of autophagy inhibitors, such as wortmannin and 3-MA, effectively hindered the formation of NETs (148, 165). Furthermore, the study found that pretreatment of neutrophils with low-molecular-weight heparin (LMWH) *in vitro* significantly impaired their ability to initiate autophagy. In a separate study involving healthy volunteers, a single administration of parnaparin as a preventive measure rendered neutrophils incapable of triggering autophagy and generating NETs (148) (refer to **Table 1** for details).

**Table 1:** The approaches to targeting NETs.

Target	Parameter assessed	Content	Disease	References
1. Inhibition of NETs				
PAD4 inhibitors				
GSK199	Western blot and H3Cit imaging assays	Inhibit activity of PAD4 subsequently lead to the damage of mouse and human NET formation	–	(165)
GSK484	Western blot and H3Cit imaging assays	Inhibit activity of PAD4, subsequently lead to the damage of mouse and human NET formation	–	(165)
BMS-P5	Western blot and fluorescent microscopy	Block formation of NETs and delays progression of multiple myeloma	Multiple myeloma	(166)
NE inhibitors				
Sivelestat	Intravital video microscopy and ELISA	Primary tumors induce NETs with targetable metastasis-promoting effects, and blocking NETosis with sivelestat significantly inhibits spontaneous metastasis to the lung and liver.	Advanced esophageal, gastric, and lung cancer. Cancer-associated liver metastasis	(133)
Antibody				
tACPA	FACS analysis and IF microscopy	tACPA reduced NET release and potentially initiated NET uptake by macrophages *in vivo*	IA, IBD, pulmonary fibrosis, and sepsis.	(167)
2. Degradation of NETs				
DNase 1	Western blot, ELISA and IF staining	Inhibition of NETs generation by DNase 1 effectively abrogated the NETs-aroused tumor growth and metastasis.	Hepatocellular carcinoma; NASH thrombosis;	(37, 115, 117, 119, 158)
DNase 1 and aspirin/hydroxychloroquine	Serum MPO-DNA level, H3Cit and Ly6G imaging assays	A combination of DNase 1 with aspirin/hydroxychloroquine effectively reduced HCC metastasis in mice model.	Hepatocellular carcinoma	(36)
3. NETs and others				
NETs and CTLs	Immunohistochemistry and flow cytometry	NETs inhibit immune cell cytotoxicity by impeding contact with tumor cells	Human cancer	(147)
NETs and acidic TME	Western blot and immunohistochemistry	Injectable adhesive hemostatic gel with tumor acidity neutralizer and NETs lyase for enhancing adoptive NK cell therapy prevents post-resection recurrence of HCC	Hepatocellular carcinoma	(146)
Autophagy inhibitors				
3-MA	Western blot, flow cytometry and scanning electron microscopy	Inhibition of autophagy by 3-MA alleviated the ROS burst and subsequent NETosis caused by diphenyl phosphate	-	(151, 168)
Wortmannin	ELISA, flow cytometry and confocal microscopy	Using of wortmannin can prevent NET generation	-	(151, 168)
NETs and LMWH	ELISA, flow cytometry and confocal microscopy	LMWH prevent the induction of autophagy of activated neutrophils and the formation of NETs	-	(151)

## 4 Discussion, concluding marks and key questions

### 4.1 NETs in other cancers

The recent advancement in our comprehension of cancer-associated NET formation is the identification of the ability of carcinomas to enhance neutrophil production of thrombogenic NETs. This discovery was first reported in 2012 (115). Neutrophil extracellular traps have now been associated with various non-infectious diseases, such as cancer (166), diabetes (169), autoimmunity (167), cardiovascular disease (168), and systemic lupus erythematosus (170), all of which have an inflammatory component. In the following section, we present and review the current understanding of cancer-associated NET formation, including the diverse mechanisms underlying NET formation in different types of cancers.

Despite significant advancements in our understanding of the mechanism of NETs in tumors, they still remain enigmatic. In 2013, a groundbreaking discovery was made, demonstrating the correlation between NET formation and malignancy. This finding suggested that the presence of intratumoral NETs may be associated with an unfavorable prognosis in a subset of patients diagnosed with Ewing sarcoma (171). Subsequently, the potential of NETs has been extensively investigated across diverse malignancies, revealing their oncogenic effect in the majority of cancers, although not in all conditions. Notably, mice with tumors exhibited an increase in plasma levels of NET formation compared to control mice. Neutrophils in this context tended to spontaneously form NETs, leading to thrombus formation and a pro-coagulant state (115, 172). Moreover, it was found that tumor cells stimulated the formation of NETs, thereby promoting breast cancer lung metastasis (172).

Low-density neutrophils (LDNs), which are a subset of activated neutrophils with distinct phenotypic and functional characteristics, exhibit differences compared to normal-density neutrophils (NDNs) in human peripheral blood polymorphonuclear neutrophils (PMNs) (173). In a murine model of orthotopic pancreatic adenocarcinoma, suppression of PAD4 resulted in decreased levels of circulating NET formation, leading to reduced tumor growth and improved survival rates. Furthermore, NETs stimulated pancreatic stellate cells, which were found to promote tumor proliferation (174). Similar findings were observed in an experimental melanoma model, where NETs accumulated in the TME and promoted cancer growth (175). Interestingly, the researchers also observed the presence of NETs in ulcerated tissue of melanoma patients, which were negatively associated with the patients’ prognostic outlook. However, co-incubation of NET formation and melanoma cells disrupted melanoma cell migration and viability (176). This observation suggests that the potential factors *in vivo* may block the anti-tumor effects of NETs in melanoma, and further exploration in this regard could be a promising direction. While the presence of tumor-associated and circulating NETs has been linked to a worse prognosis in patients with diffuse large B-cell lymphoma, the exact correlation remains to be fully elucidated in humans. Both *in vivo* and *in vitro* studies have also demonstrated that NETs promote the proliferation and migration of tumor cells (177).

In recent studies by Yang et al., the role of NETs in promoting cancer metastasis, as well as the association between NETs and neutrophil DNA, has been strongly supported (17). They investigated specific biomarkers, such as MPO and H3Cit, to identify distinct neutrophils and their associated NETs. The results revealed that liver metastases exhibited the highest level of NET infiltration. Interestingly, higher levels of serum MPO-DNA were found to be independently associated with subsequent liver metastasis, while no such correlation was observed in other organs. These findings suggest that excessive hepatic NET formation may occur prior to the detection of metastases in breast cancer patients, thereby promoting subsequent hepatic metastasis (17). Furthermore, experiments conducted on PAD4-deficient C57BL/6 mice demonstrated a significant attenuation in the formation of NETs and hepatic metastases. Conversely, NET-DNA extracted from PMA-treated neutrophils exhibited an opposite effect. Additionally, tumor cells exhibited enhanced chemotaxis towards the DNA component of hepatic NETs through high-affinity binding with a transmembrane protein called CCDC25, which is expressed on carcinoma cells. The interaction between NET formation DNA and CCDC25 promotes cancer cell migration and initiates metastasis by triggering intracellular signaling pathways (17).

It is evident that the formation of NETs plays a crucial role in cancer metastasis, particularly in the context of liver metastases. Targeting the components of hepatic NETs, such as MPO and H3Cit, may hold significant therapeutic potential for the treatment of HCC. Moreover, exploring the interplay between immune cells and cancer cells, as well as the potential of injectable hydrogels in enhancing immunotherapy, and the impact of senescence-associated secretory phenotype (SASP) on the tumor microenvironment, could provide valuable insights for future research. Ultimately, by targeting heparin as a potential therapeutic intervention and modulating the tumor immune microenvironment through the interaction of cancer cells and immune cells, novel strategies for HCC treatment may be developed.

Metastasis, the spread of cancer cells to distant tissues, is the leading cause of mortality in cancer patients, surpassing the impact of the original primary tumor. The process of metastatic initiation can persist for an extended period, potentially due to the protective effects of immune cells or inadequate angiogenic capacity. This leads to a state of uncertainty between cell proliferation and apoptosis (10, 12, 178). The factors that disrupt this delicate balance remain elusive. D. Barkan and B.L. Pierce proposed an intriguing theory suggesting that low-grade inflammation may trigger the transition from dormancy to proliferation in metastatic cells (179, 180). Recently, a study found that the recruitment and activation of neutrophils occur prior to treatment with LPS in rats with dormant lung tumor cells (142, 181). Depleting neutrophils eliminated the awakening of dormant cancer cells induced by LPS. The presence of NETs in the lung was rapidly (within 4 hours) induced by LPS instillation and persisted for 24 hours. Similar observations were made using a PAD4 inhibitor. These findings were also confirmed in a prostate cancer model exposed to tobacco smoke, a proinflammatory stimulus. Therefore, various rat models exposed to multiple inflammatory stimuli induce the awakening of dormant cancer cells through NET formation. The authors also discovered that the reactivation of quiescent carcinoma cells relies on ECM remodeling and laminin cleavage, which require matrix metallopeptidases 9 (MMP9) and NE, an enzyme associated with NETs. The appearance of a novel epitope in laminin, resulting from proteolytic remodeling, promotes cancer cell proliferation through the α3β1 integrin pathway. The presence of NETs prompts quiescent cancer cells to initiate proliferation. However, when these disseminated cells embark on their proliferation journey, they become susceptible to recognition by T cells and NK cells (178, 182, 183). The specific impact of the adaptive immune system in mouse models remains unclear. Nevertheless, lipopolysaccharide exposure elevates glucocorticoid levels (184)while simultaneously suppressing adaptive immune cells. Consequently, the combination of lipopolysaccharide and smoking may facilitate the growth of dormant cells. This is achieved by triggering signaling pathways through NETs to initiate proliferation and concurrently disrupting immune control through glucocorticoids.

### 4.2 Conclusions

Over the past decade, the crucial role of neutrophils in both inflammatory and non-inflammatory diseases has been firmly established. One of the contributing factors is NET. In the context of cancer, particularly HCC, NET formation may play a significant role in disease progression. However, studying NETs presents challenges, as primary human neutrophils cannot be transfected, and inhibiting NET formation pathways *in vivo* is difficult. Furthermore, the heterogeneity between human and mouse neutrophils raises questions about the relevance of NET formation in tumors, as most studies rely on mouse models (13, 98, 185–187).

Neutrophils can polarize into two subtypes: N1, which exhibits anti-tumorigenic properties (188, 189), and N2, which displays pro-tumorigenic characteristics (190–192). Both subtypes have the capacity to generate NETs. Additionally, other matrix cells in the TME, such as basophils (193–195), eosinophils (27, 195–198), mast cells (199, 200), and macrophages (201), can also release extracellular DNA traps. However, the specific components of NETs or extracellular DNA traps that possess anti-tumorigenic or pro-tumor effects remain unclear. Therefore, it is crucial to inhibit NET formation without compromising other effective constituents or generating detrimental degradation products of neutrophil extracellular traps. This requires a comprehensive understanding of the mechanisms involved in NET formation and degradation. Furthermore, targeting NETs and related enzymes may hold promise as a therapeutic strategy for combating cancer metastasis.

The concentration of NETs is elevated in both blood serum and tissue specimens from patients with HCC at different stages of liver cancer, including HCC patients with HBV, those who have undergone liver surgery (including liver IRI), NASH, and liver cirrhosis. While most research has focused on the role of NETs in regulating tumor progression and proliferation, further investigation is needed to understand the specific components of NETs that interact with signaling pathways. Additionally, more research is required to identify the mechanisms by which NETs are regulated *in vivo*. Although previous *in vivo* or *in vitro* studies have primarily induced NET formation using LPS or PMA, the detailed mechanisms underlying the interplay between NETs and other immune cell components, such as macrophages, CD4^+^ T cells, and Tregs, remain largely unexplored. Moreover, various risk factors associated with disease progression, including diabetes, age, and the presence of advanced cirrhosis or fibrosis, have been identified through clinical research. However, there are still gaps in our understanding of the pathobiology of HCC.

The formation of NETs has been identified as a valuable biomarker for diagnostic, predictive, and prognostic purposes in various human cancers, including HCC. Abundant evidence supports the notion that tumor-associated NET formation plays a pivotal role in facilitating the unchecked proliferation of malignant cells, creating a permissive environment for cancer development, expediting tumor progression during systemic infection, and promoting cancer-associated thrombosis in HCC. Furthermore, the presence of NETs in HCC patients has been shown to enhance metastatic potential and contribute to disease recurrence. The potential of NETs as therapeutic targets for HCC holds great promise, as targeting NETs could potentially prevent the progression of liver cancer, inhibit metastasis, mitigate cancer-associated thrombosis, and reduce post-operative recurrence. Clinical trials investigating anti-NET therapies, either alone or in combination with immune checkpoint inhibitors (ICI), are currently underway. It is hoped that combination therapy will yield a synergistic effect, effectively treating HCC patients by counteracting NET-mediated immunosuppression in the presence of ICI.

### 4.3 Key questions for further research

**Figure 6:**
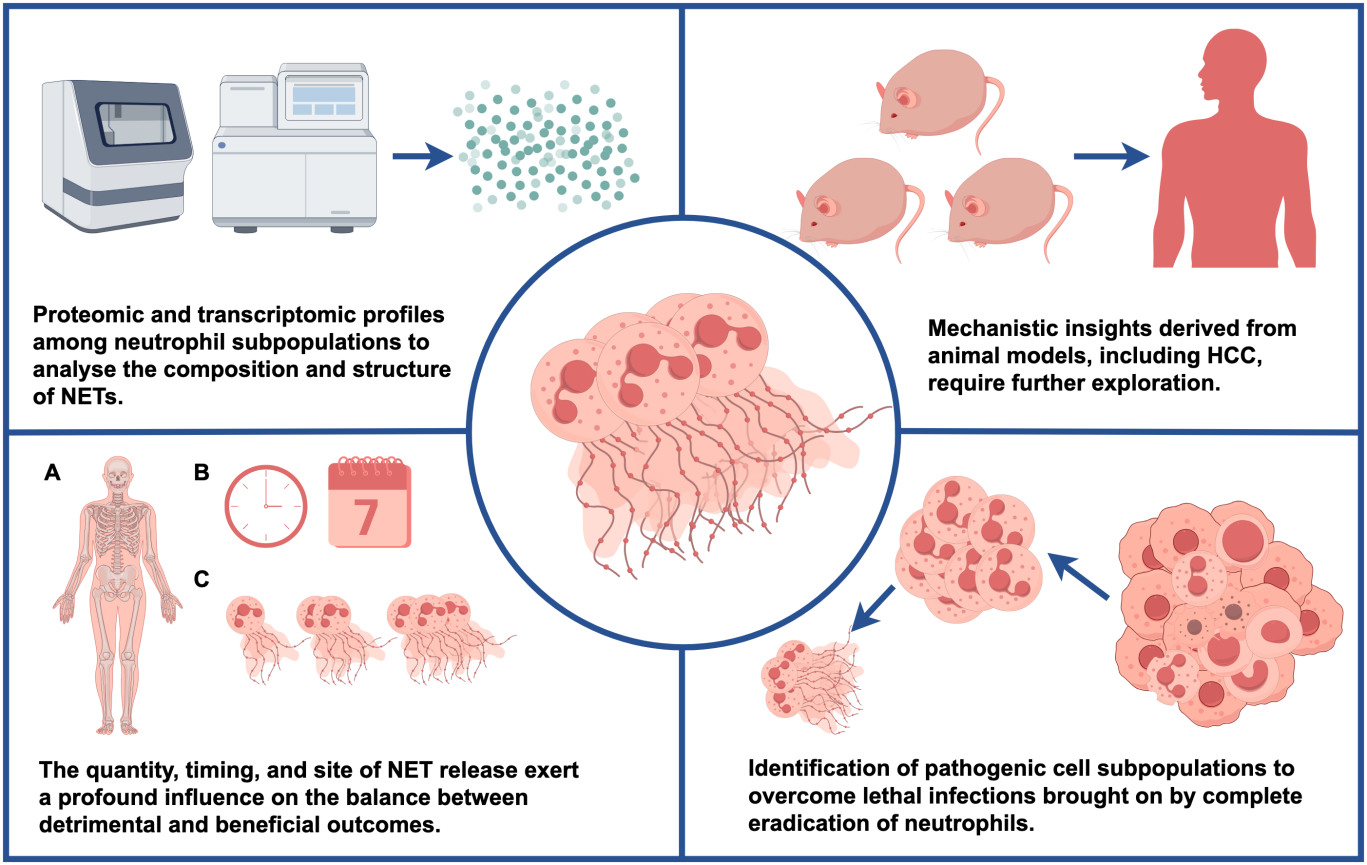
This figure illustrates potential future research directions and key questions.

It is necessary to determine the immunomodulatory properties of NETs, such as their antibacterial activity, which play a crucial role in triggering or suppressing inflammation, in order to further elucidate their mechanisms. Conversely, changes in the composition and structure of NETs may result from variations in proteomic and transcriptomic profiles among neutrophil subpopulations, as well as activation by diverse stimuli. These aspects warrant further investigation. Moreover, the quantity, timing, and site of NET release exert a profound influence on the balance between detrimental and beneficial outcomes. Given the inherent heterogeneity of neutrophils within the hepatocellular carcinoma TME (11), exploring the involvement of distinct neutrophil subpopulations in the timing and quantity of NET release within the context of hepatocellular carcinoma (secondary dysregulation induced by disease initiation and other factors) may represent a promising research avenue. Furthermore, NETs have been identified in various human diseases, prompting the need to investigate their potential utility as markers of therapeutic efficacy. Additionally, further investigate the mechanisms observed in animal models, including those related to HCC, and determine their applicability and relevance in clinical settings is crucial. Nonetheless, it is worth noting that most current NET research remains mechanistic in nature, with clinical translation remaining a distant goal. Nevertheless, a more comprehensive understanding of the molecular mechanisms underlying NETs holds the potential to bridge this gap. Lastly, as strategies for neutrophil clearance are often unviable in the context of most human conditions, the field faces the challenge of identifying pathogenic cell subpopulations that can be effectively targeted during therapy (**Figure 6**).

## Author contributions

WXZ: Data curation, Writing – original draft, Writing – review & editing, Conceptualization, Formal Analysis, Investigation, Software, Validation. CF: Writing – original draft. SD: Conceptualization, Investigation, Software, Writing – original draft. XL: Methodology, Supervision, Writing – original draft. HC: Data curation, Writing – original draft. WCZ: Writing – review & editing.
